# Association between serum erythropoietin levels and renal reversibility in patients with renal impairment from multiple myeloma

**DOI:** 10.1002/cam4.3050

**Published:** 2020-04-20

**Authors:** Hiroki Kobayashi, Toshiki Terao, Takafumi Tsushima, Yoshiaki Abe, Daisuke Miura, Kentaro Narita, Akihiro Kitadate, Masami Takeuchi, Kosei Matsue

**Affiliations:** ^1^ Division of Hematology/Oncology Department of Medicine Kameda Medical Center Kamogawa‐shi Chiba‐Ken Japan

**Keywords:** erythropoietin, multiple myeloma, renal reversibility

## Abstract

Renal impairment (RI) is a common clinical presentation in patients with multiple myeloma (MM). Despite treatment with novel agents or management strategies that focus on the disease response, some patients develop irreversible RI. This study aimed to determine the influencing, clinical variables of renal reversibility in patients with RI treated with novel drugs. We analyzed 244 patients newly diagnosed with MM retrospectively. Maximum renal response was assessed according to the criteria for the definition of renal response proposed by the International Myeloma Working Group. Major renal response was defined as the occurrence of complete renal response or partial renal response. RI (a glomerular filtration rate < 50 mL/min/1.73 m^2^) was observed in 110 patients (45%). In total, 56 patients (51%) achieved a major renal response. Serum erythropoietin (EPO) levels >25 mIU/mL (*P* < .001) and a percentage of urinary albumin excretion ≤25% (*P* < .001) were both significant factors that influenced the occurrence of major renal responses. Both remained significant factors associated with renal reversibility in the multivariate analysis. Patients were assigned a score of 1 each for EPO >25 mIU/mL and urinary albumin ≤25%. The estimated 6‐month rates of major renal responses of patients with scores of 2, 1, and 0 were 78.6%, 30.6%, and 0%, respectively (*P* < .001). In conclusion, a serum EPO level >25 mIU/mL is an independent predictive factor for major renal response and may predict renal reversibility more accurately when urinary albumin level is congruently ≤25%.

## Graphical abstract

Renal impairment (RI) is a common complication of multiple myeloma (MM), and this study aimed to determine the influencing, clinical variables of renal recovery in RI patients treated with novel drugs. Serum erythropoietin (EPO) levels >25 mIU/mL and percentage of urinary albumin ≤25%, were both significantly associated with a shorter time to major renal response. Serum EPO level >25 mIU/mL is an independent positive predictive factor for major renal response and predict renal reversibility more accurately when urinary albumin level is congruently ≤25%.

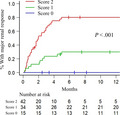

## 1 INTRODUCTION

Renal impairment (RI) is one of the most common clinical presentations in patients with multiple myeloma (MM) and contributes to its morbidity and mortality. Approximately 40% of MM patients present with RI at the diagnosis; 10% of these will require dialysis.^1^, ^2^, ^3^, ^4^, ^5^ Patients with RI generally have a worse prognosis than those without.^1^, ^6^, ^7^, ^8^ Toxic monoclonal free light chains (FLC) contribute to RI in those with MM through different mechanisms. The main manifestation of FLC‐associated kidney injury is cast nephropathy (CN). Toxic FLC also leads renal amyloidosis and monoclonal immunoglobulin deposition disease.^9^, ^10^ Age‐related comorbidities, including hypertension and diabetes, can also contribute to renal dysfunction.^11^

Previous studies have shown that rapid removal of FLC contributes to renal recovery.^12^, ^13^, ^14^ Novel drugs, such as proteasome inhibitors and immunomodulatory drugs, help in the rapid myeloma response, leading to the recovery of kidney function.^3^, ^15^, ^16^, ^17^ In addition, the reversibility of RI partly depends on the pathogenesis of the kidney injury. In CN patients, the removal of toxic FLC was related to the renal recovery.^18^ Earlier reports have additionally demonstrated that the proportion of urinary albumin was lower in patients with CN, while it was higher in patients with renal amyloidosis.^11^, ^19^ In relation to these results, we previously reported that the percentage of urinary albumin excreted reflects the pathogenesis of RI and predicted renal response in myeloma patients.^20^

However, some patients continue to develop severe RI, despite novel anti‐myeloma therapy and the achievement of early FLC reduction. In patients receiving novel agents, an additional factor, which can indicate the reversibility of RI, remains to be determined. Therefore, the purpose of this study was to evaluate the clinical factors that influenced renal reversibility in patients with RI who received novel anti‐myeloma therapy.

## 2 METHODS

We reviewed the clinical record of patients with newly diagnosed MM at our institute between January 2008 and December 2018. All patients received novel agents including proteasome inhibitors and immunomodulatory drugs as initial therapy. In this study, RI was defined as an estimated glomerular filtration rate (eGFR) at the time of diagnosis lower than 50 mL/min/1.73 m^2^ to assess renal recovery. An eGFR was evaluated according to the simplified Modification of Diet in Renal Disease formula.^21^ Maximum renal response was assessed using the criteria for the definition of renal response proposed by the International Myeloma Working Group as follows^4^: renal complete response (CRrenal) was defined as a sustained improvement from an eGFR baseline value of lower than 50 to ≥60 mL/min/1.73 m^2^. Partial renal response (PRrenal) was defined as a sustained recovery of eGFR at the time of diagnosis of less than 15 to 30‐59 mL/min/1.73 m^2^; a minor renal response (MRrenal) was defined as a sustained improvement of baseline eGFR from less than 15 to 15‐29 mL/min/1.73 m^2^ or improvement of eGFR at the time of diagnosis from 15‐29 mL/min/1.73 m^2^ to 30‐59 mL/min/1.73 m^2^. In this study, the definition of a major renal response was the achievement of CRrenal or PRrenal. Kidney biopsies were performed in some patients to evaluate the cause of RI and nephropathologists assessed the biopsy samples. We focused the analysis on the major renal response, as this degree of renal recovery is clinically more relevant.^15^, ^17^

We used Student's *t* test or the Mann‐Whitney *U* test to compare the continuous variable average values and Fisher's exact test to compare the proportion of categorical variables between groups. We used the receiver operating characteristic (ROC) to determine an adequate cutoff level to predict the major renal response.^22^ Time to major renal response was defined as the time from the date of diagnosis to the date of first major renal response occurrence. Patients lost to follow‐up or those who died before achieving a major renal response were considered as censored events. The Kaplan‐Meier method was performed to evaluate time to major renal response, and groups were compared using a log‐rank test. Univariate and multivariate analyses of time to major renal response were conducted using Cox proportional hazards regression models. Factors with a *P* < .10 in the univariate analysis were included in the multivariable models. All *P* values were two‐sided. The threshold for statistical significance was a *P* < .05. All the analyses were conducted with EZR version 1.37. ^23^

The patients or their families provided written informed consent. The study approval was obtained from the institutional review board of our institute, and the study was conducted according to the tenets of the Declaration of Helsinki.

## 3 RESULTS

### 3.1 Patient characteristics

**FIGURE 1:**
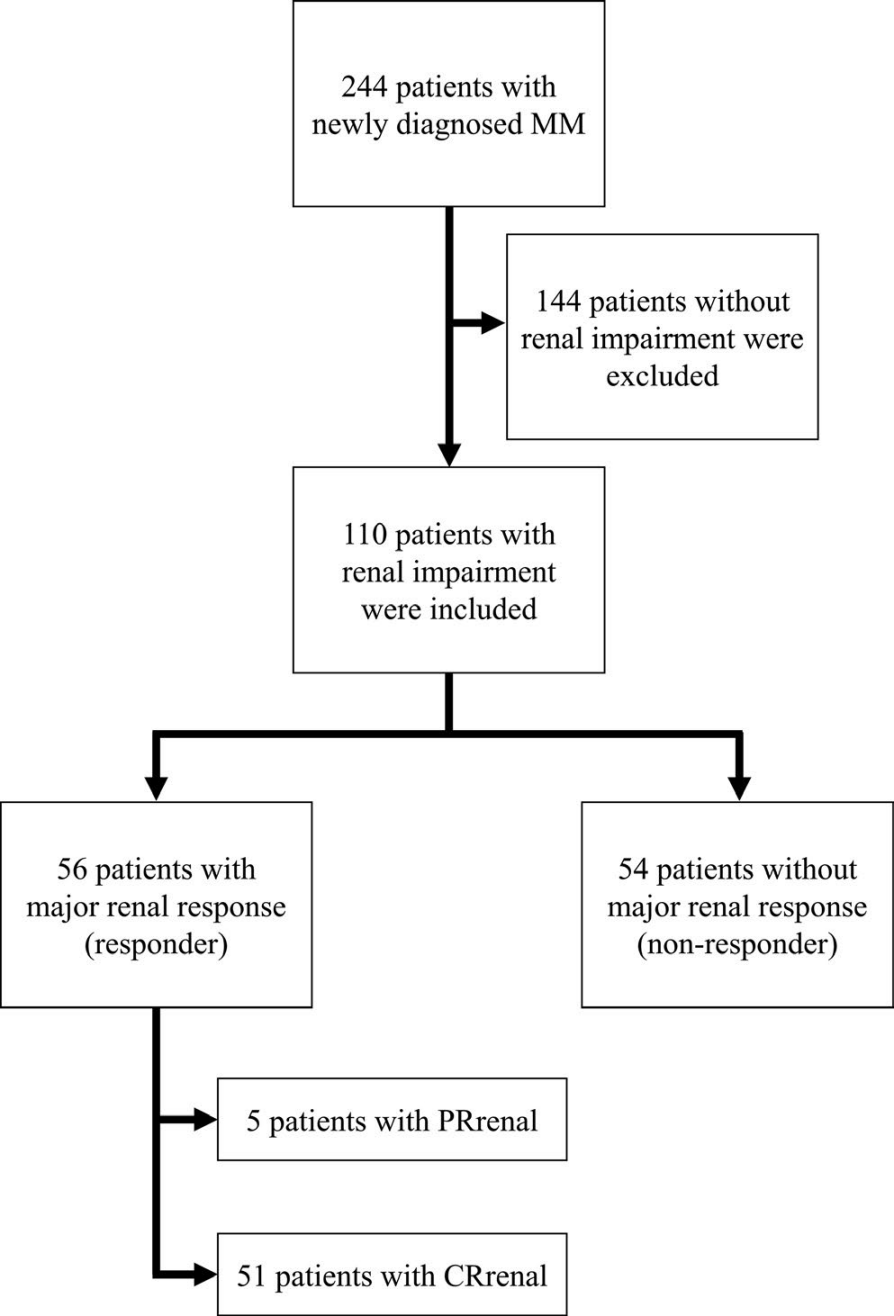
Patient flow chart

Of the 244 consecutive patients with MM admitted at our institute, we enrolled 110 (45.1%) patients with RI in the present study. The patients were a median age of 72 years. Thirty‐one (28.2%) had light‐chain only isotypes; 59.1% had kappa light chains. Improvement in RI was observed in 79 (71.8%) patients. A major renal response was achieved by 56 (50.9%) patients, of these, 5 (4.5%) achieved PRrenal and 51 (46.4%) achieved CRrenal. Patients who achieved a major renal response were defined as responders (Figure 1).

The baseline characteristics of responders and non‐responders are shown in Table 1. The serum erythropoietin (EPO) levels at baseline were available for 97 (88.2%) patients with RI. Of these, 47/56 (83.9%) had a major renal response and 50/54 (92.6%) did not. There were significant differences in age and baseline serum calcium level between responders and non‐responders. Renal function at the diagnosis was more significantly declined in non‐responders than in responders (median eGFR, 22.3 in non‐responders vs 34.1 in responders; *P* < .001). There were no significant differences in involved FLC (iFLC) levels at baseline, percentages of iFLC reduction, and the rate of myeloma response between responders and non‐responders. The median percentage of urinary albumin excretion was significantly lower (8.7% in responders vs 26.0% in non‐responders; *P* = .002), while the median level of EPO was significantly higher in responders (51.1 in responders vs 17.1 in non‐responders; *P* < .001).

**TABLE 1:** Comparison of the baseline characteristics and laboratory variables between the renal responder and non‐responder groups

	Non‐responders	Responders	*P* value
	(n = 54)	(n = 56)	
Age (y), median (IQR)	77.5 (70.3‐84.0)	70.5 (64.0‐77.8)	0.001
Male sex, n (%)	24 (44.4)	29 (58.0)	0.18
Light chain only, n (%)	14 (25.9)	17 (30.4)	0.67
Kappa light chain type, n (%)	32 (59.3)	33 (58.9)	1.0
Hemoglobin (g/dL), median (IQR)	8.9 (7.7‐9.8)	8.4 (7.7‐9.9)	0.42
LDH (U/L), median (IQR)	190 (159‐231)	193 (151‐293)	0.64
Creatine (mg/dL), median (IQR)	2.14 (1.42‐4.32)	1.40 (1.17‐2.26)	0.012
eGFR (mL/min/1.73 m^2^), median (IQR)	22.3 (9.3‐33.6)	34.1 (19.8‐43.6)	<0.001
Cystatin‐C (mg/dL), median (IQR)^a^	2.32 (1.73‐3.12)	1.85 (1.43‐2.65)	0.007
Calcium (mg/dL), median (IQR)	9.6 (9.3‐10.2)	10.2 (9.5‐12.3)	0.003
Albumin (g/dL), median (IQR)	3.3 (2.8‐3.8)	3.2 (2.5‐3.7)	0.39
β2‐microglobulin (mg/dL), median (IQR)	8.2 (5.3‐13.2)	8.3 (6.0‐13.4)	0.80
ISS stage 3, n (%)	41 (75.9)	46 (82.1)	0.49
Revised ISS stage 3, n (%)^b^	14 (27.5)	28 (53.8)	0.009
Myeloma response VGPR, n (%)	35 (66.0)	40 (71.4)	0.53
iFLC (mg/dL), median (IQR)	2625 (246‐10285)	2585 (437‐5700)	0.65
%iFLC reduction at day 21 (%), median (IQR)^c^	85.7 (65.3‐94.8)	89.9 (72.8‐98.1)	0.29
Percentage of urinary albumin excretion (%), median (IQR)^d^	26.0 (5.2‐51.9)	8.7 (4.9‐18.1)	0.002
Erythropoietin (mIU/mL), median (IQR)^e^	17.1 (12.2‐27.4)	51.1 (27.0‐88.1)	<0.001

Note

Abbreviations: eGFR, estimated glomerular filtration rate; iFLC, involved free light chain; IQR, interquartile range; ISS, international staging system; LDH, lactate dehydrogenase; R‐ISS, revised international staging system; VGPR, very good partial response.

^a^ n = 99 (non‐responder, n = 51; responder, n = 48).

^b^ n = 103 (non‐responder, n = 51; responder, n = 52).

^c^ n = 97 (non‐responder, n = 49; responder, n = 48).

^d^ n = 105 (non‐responder, n = 51; responder, n = 54).

^e^ n = 97 (non‐responder, n = 50; responder, n = 47).

### 3.2 Prognostic factors for major renal response

The median value of EPO in responders was significantly higher than that in non‐responders (Table 1; *P* < .001). As serum EPO levels are physiologically regulated by the degree of hemoglobin concentration, the presence of anemia is necessary for inducing an increase in EPO production.^24^ Thus, six patients (three non‐responders and three responders) without anemia (ie, men and women with Hb > 12.0 g/dL and >11.0 g/dL, respectively) were excluded from further analyses. The optimal cutoff for EPO level was determined via ROC analysis and was determined to be 24.6 mIU/mL. This had a specificity of 0.711, sensitivity of 0.884, and area under the curve (AUC) of 0.829 with 95% confidence intervals (CI) of 0.739‐0.919. Accordingly, an EPO level of 25 mIU/mL was used as the cutoff.

In addition, we examined whether albuminuria and the degree of iFLC reduction contributed to a major renal response. Using ROC analysis, the cutoff value for the percentage of urinary albumin excreted was 25% with a specificity of 0.510, sensitivity of 0.907, and AUC of 0.672; a percentage of iFLC reduction on day 21 was 85%, with a specificity of 0.531, sensitivity of 0.604, and AUC of 0.563.

**FIGURE 2:**
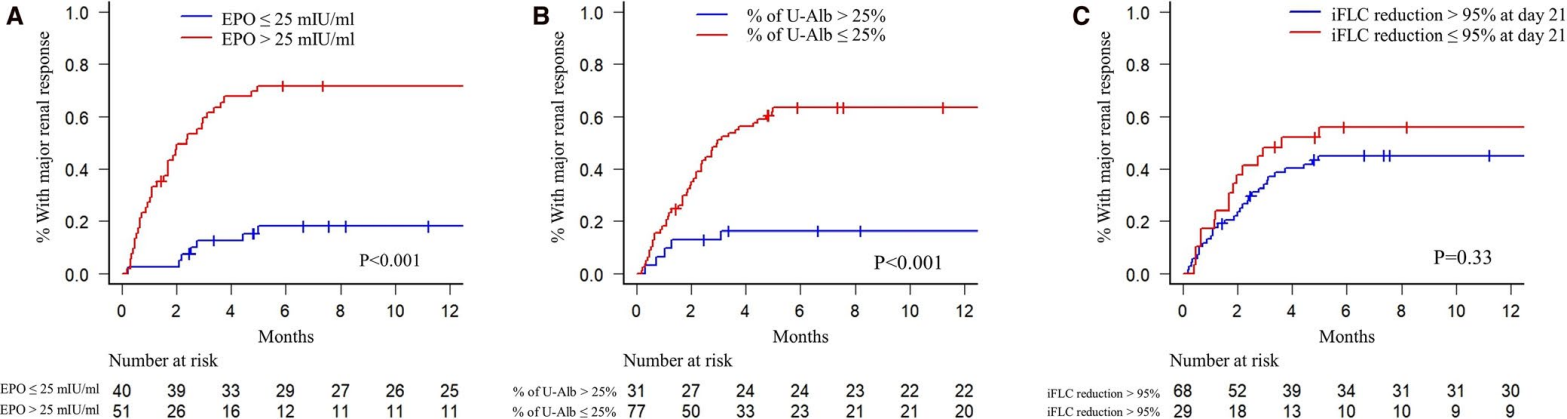
Kaplan‐Meier curve for time to major renal response according to (A) the level of serum erythropoietin, (B) percentage of urinary albumin excretion, and (C) percentage of iFLC reduction at day 21. EPO, erythropoietin; iFLC, involved free light chain

The patients with an EPO level >25 mIU/mL at baseline had a significantly shorter time to major renal response (2.4 months vs not estimated [NE], *P* < .001). The estimated rate of major renal response at 6 months was 70.4% (95% CI, 57.7‐82.2) and 19.6% (95% CI, 9.3‐35.2) in patients with an EPO level >25 mIU/mL and in those with an EPO level ≤25 mIU/mL, respectively (Figure 2A). The percentage of urinary albumin excreted (≤25%) was also significantly associated with a shorter time to major renal response (3.0 months vs NE, *P* < .001). The estimated 6‐month major renal response was 63.5% (95% CI, 52.7‐74.3) in patients with urinary albumin ≤ 25%, compared with 16.3% (95% CI, 7.1‐34.7) in those with a urinary albumin percentage >25% (Figure 2B). The patients with a reduction in iFLC on day 21 of over 85% tended to have a more rapid median time to major renal response, although this was not significant (5.0 months vs 51.6 months, *P* = .49) (Figure 2C).

From these results, we hypothesized that the baseline EPO and urinary albumin could be strong predictive factors for achievement of major renal response. Multivariate analysis revealed that an EPO level >25 mIU/mL (HR, 5.99; 95% CI, 2.38‐15.1; *P* < .001) and urinary albumin excretion ≤ 25% (HR, 6.67; 95% CI, 1.91‐23.3; *P* = .003) were independent predictors for rapid achievement of major renal response when adjusted for age, eGFR, and calcium at the time diagnosis (Table 2).

**TABLE 2:** Univariate and multivariate analyses of variables that influenced time to major renal response

	Univariate			Multivariate		
	HR	95% CI	*P* value	HR	95% CI	*P* value
Age < 75 y	2.56	1.45‐4.56	0.001	6.96	3.08‐15.7	<0.001
eGFR > 30 mL/min/1.73 m^2^	2.33	1.37‐3.95	0.002	2.58	1.27‐5.23	0.009
Ca > 10.5 mg/dL	2.47	1.45‐4.19	<0.001	5.64	2.67‐11.9	<0.001
% of urinary albumin excretion ≤ 25%^a^	5.61	2.23‐14.1	<0.001	6.67	1.91‐23.3	0.003
Erythropoietin > 25 mIU/mL^b^	6.45	2.86‐14.5	<0.001	5.99	2.38‐15.1	<0.001
Male sex	1.34	0.78‐2.76	0.29			
ISS stage 3	1.27	0.64‐2.52	0.50			
Myeloma response VGPR	1.09	0.60‐1.97	0.79			
iFLC reduction > 85% at day 21^c^	1.23	0.69‐2.19	0.49			

Abbreviations: Ca, calcium; CI, confidence interval; eGFR, estimated glomerular filtration rate; HR, hazard ratio; iFLC, involved free light chain; ISS, international staging system; VGPR, very good partial response.

^a^ n = 105.

^b^ n = 91.

^c^ n = 102.

### 3.3 Development of a predictive score for major renal response

As factors correlated to major renal response included serum EPO level and percentage of urinary albumin, we examined the combination of the two factors as a predictor of major renal response. In 91 patients, data were available for analysis on both variables.

**FIGURE 3:**
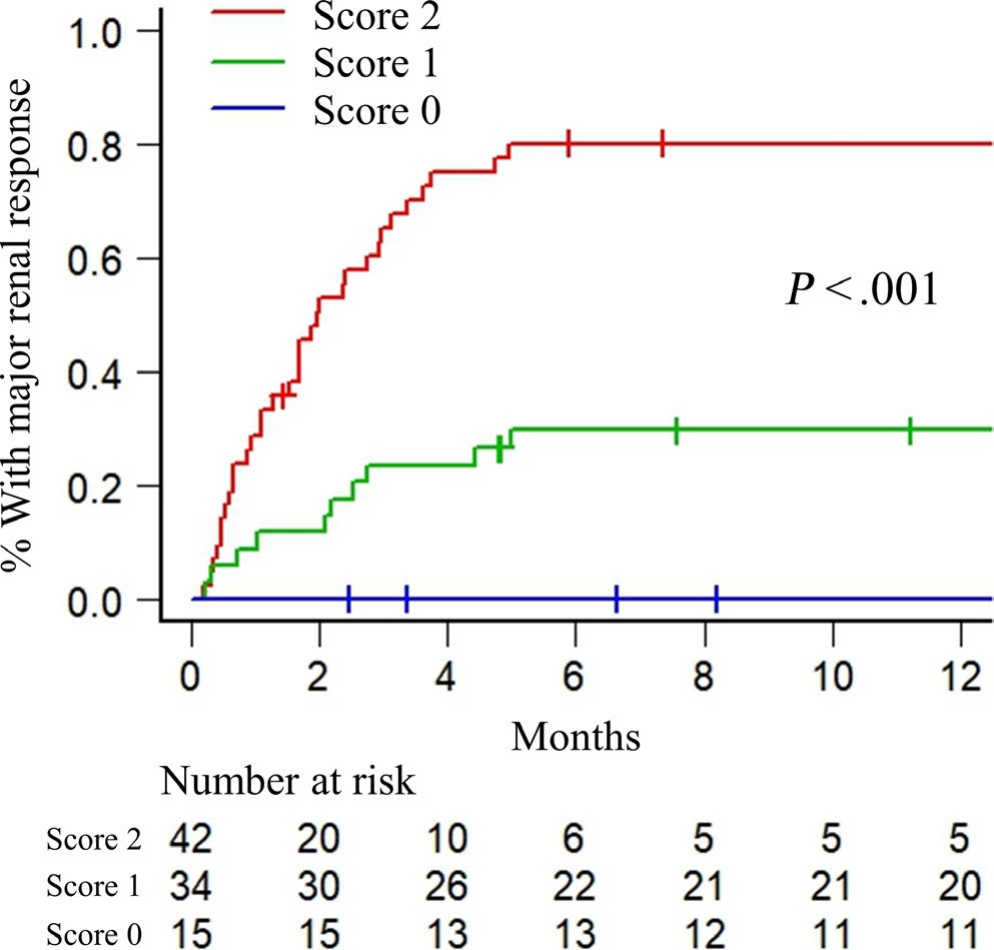
Kaplan‐Meier curve for time to major renal response according to the scoring system; thresholds for serum erythropoietin and percentage of urinary albumin excretion were > 25 mIU/mL and ≤ 25%, respectively (score of 2, both erythropoietin > 25 mIU/mL and urinary albumin ≤ 25%; score of 1, either erythropoietin > 25 mIU/mL or urinary albumin ≤ 25%; score of 0, neither erythropoietin > 25 mIU/mL nor serum albumin ≤ 25%)

We assigned a score of 1 for each of the two variables (serum EPO > 25 mIU/mL and urinary albumin excretion ≤ 25%) to divide patients into three groups. In total, there were 42 (46.2%), 34 (37.3%), and 15 (16.5%) patients with scores of 2, 1, and 0, respectively. The median time to major renal response of patients with scores of 2, 1, and 0 was significantly different (2.0 months vs NE vs NE, *P* < .001). The estimated 6‐month rates of major renal response of patients with scores of 2, 1, and 0 were 78.6%, 30.6%, and 0%, respectively (Figure 3).

### 3.4 Pathological findings according to EPO

Next, we evaluated the association between renal pathology and the level of serum EPO. In our study, 23 patients (21.0%) underwent kidney biopsy. Among them, 17 (7 patients with EPO > 25 mIU/mL and 10 patients with EPO ≤ 25 mIU/mL) had EPO data available.

Approximately 80% of patients with and without EPO levels >25 mIU/mL were diagnosed as CN. Concurrent tubular amyloidosis was found in two patients in the low EPO level group. However, renal interstitial fibrosis and tubular atrophy were observed in 70% of patients with EPO levels ≤25 mIU/mL, whereas approximately 30% of patients with EPO > 25 mIU/mL had renal interstitial fibrosis and tubular atrophy.

## 4 DISCUSSION

Renal impairment is a common clinical presentation in patients with newly diagnosed MM.^25^ Novel agents enable patients with myeloma to achieve renal function recovery.^15^ However, a considerable number of patients who achieve early and significant reduction of iFLC do not attain major renal responses. Thus, it is important to detect factors associated with major renal recovery other than early myeloma response. The current study confirms our previous finding that MM patients with RI who had a low level of urinary albumin tend to have a high probability of resolved RI and showed an association between baseline serum EPO levels and the reversibility of kidney function.

Erythropoietin is an erythropoietic glycoprotein hormone mainly produced by specific cells in the kidney interstitium.^26^ According to recent evidence, renal EPO‐producing cells and renal myofibroblasts differentiated from the same embryonic cells, and inflammatory signaling caused the phenotypic transition of renal EPO‐producing cells to myofibroblasts.^27^, ^28^ Thus, interstitial fibrosis is the main cause of reduced EPO production.^29^ In addition, the degree of renal interstitial fibrosis and tubular atrophy was more strongly correlated with impaired kidney function than structural changes in the glomeruli; furthermore, interstitial fibrosis is regarded as an index of renal functional impairment.^30^, ^31^ Therefore, serum EPO levels are associated with the extent of renal interstitial fibrosis, and EPO levels reflect residual kidney function. In diabetes mellitus, Fujita et al showed that a low EPO level was also associated with faster renal function decline.^32^

The cutoff value of EPO in our study (25 mIU/mL) was close to the upper limit of the normal range (4.2‐23.7 mIU/mL). Previous reports showed that the value of serum EPO in patients with renal insufficiency was less than the upper limit despite the presence of anemia.^33^, ^34^ The cutoff value in our study was, therefore, appropriate for detecting irreversible kidney injury. MM is a unique disease in that RI and anemia occur congruently. In this regard, serum EPO levels can be used to predict renal reversibility in patients with MM if they have concurrent anemia.

We showed that the combination of EPO and urinary albumin could predict major renal responses more accurately because the percentage of urinary albumin excretion reflected the cause of RI in MM and the level of EPO reflected renal fibrosis. The categorization helps to understand the precise pathophysiology of RI in patients with MM, particularly in patients contraindicated for renal biopsy. Using this score, renal recovery can be predicted before starting anti‐myeloma therapy. Notably, none of the patients with a score of 0 achieved a major renal response (Figure 3). This indicates that this score was useful in detecting patients with completely irreversible RI.

Our study has a number of limitations. First, as a retrospective analysis, patients were treated heterogeneously. However, all patients received bortezomib or IMiD‐containing regimens as induction therapy. Second, some data on EPO were missing, and the association between EPO and renal recovery is not applicable in patients without anemia. Third, renal biopsy data were only available for a small subset of patients. Finally, the present study was a single‐center study and not validated.

In summary, we retrospectively analyzed factors that affected renal reversibility in MM. Our analysis is the first to show that EPO > 25 mIU/mL is an independent positive predictive factor for the achievement of a major renal response in patients with concurrent RI and anemia. In addition, with a combination of EPO > 25 mIU/mL and the percentage of urinary albumin excreted ≤ 25%, renal recovery could be more accurately predicted. Therefore, patients with irreversible RI were identified using our scoring system. Our findings should be validated further in large confirmatory studies.

## CONFLICTS OF INTEREST

The authors report no conflict of interest.

## AUTHOR CONTRIBUTIONS

HK, TT, and KM designed the study, collected the data, wrote the manuscript, and provided patient care. TT, YA, DM, KN, AK, and MT provided patient care. All authors reviewed and approved the manuscript.

## Data Availability

The data that support the findings of this study are available on request from the corresponding author. The data are not publicly available due to privacy or ethical restrictions.
